# Distinct high resolution genome profiles of early onset and late onset colorectal cancer integrated with gene expression data identify candidate susceptibility loci

**DOI:** 10.1186/1476-4598-9-100

**Published:** 2010-05-06

**Authors:** Marianne Berg, Trude H Ågesen, Espen Thiis-Evensen, [the INFAC-study group], Marianne A Merok, Manuel R Teixeira, Morten H Vatn, Arild Nesbakken, Rolf I Skotheim, Ragnhild A Lothe

**Affiliations:** 1 Department of Cancer Prevention, Institute for Cancer Research, Oslo University Hospital, Norwegian Radium Hospital, Oslo, Norway; 2 Centre for Cancer Biomedicine, University of Oslo, Oslo, Norway; 3 Medical Department, Oslo University Hospital, Rikshospitalet, Oslo, Norway; 4 Department of Gastrointestinal Surgery, Oslo University hospital, Aker, Oslo, Norway; 5 Faculty of Medicine, University of Oslo, Oslo, Norway; 6 Department of Genetics, Portuguese Oncology Institute, Porto, Portugal; 7 Akershus University Hospital, University of Oslo, Oslo, Norway

## Abstract

**Background:**

Estimates suggest that up to 30% of colorectal cancers (CRC) may develop due to an increased genetic risk. The mean age at diagnosis for CRC is about 70 years. Time of disease onset 20 years younger than the mean age is assumed to be indicative of genetic susceptibility. We have compared high resolution tumor genome copy number variation (CNV) (Roche NimbleGen, 385 000 oligo CGH array) in microsatellite stable (MSS) tumors from two age groups, including 23 young at onset patients without known hereditary syndromes and with a median age of 44 years (range: 28-53) and 17 elderly patients with median age 79 years (range: 69-87). Our aim was to identify differences in the tumor genomes between these groups and pinpoint potential susceptibility loci. Integration analysis of CNV and genome wide mRNA expression data, available for the same tumors, was performed to identify a restricted candidate gene list.

**Results:**

The total fraction of the genome with aberrant copy number, the overall genomic profile and the *TP53 *mutation spectrum were similar between the two age groups. However, both the number of chromosomal aberrations and the number of breakpoints differed significantly between the groups. Gains of 2q35, 10q21.3-22.1, 10q22.3 and 19q13.2-13.31 and losses from 1p31.3, 1q21.1, 2q21.2, 4p16.1-q28.3, 10p11.1 and 19p12, positions that in total contain more than 500 genes, were found significantly more often in the early onset group as compared to the late onset group. Integration analysis revealed a covariation of DNA copy number at these sites and mRNA expression for 107 of the genes. Seven of these genes, *CLC, EIF4E*, *LTBP4, PLA2G12A, PPAT*, *RG9MTD2*, and *ZNF574*, had significantly different mRNA expression comparing median expression levels across the transcriptome between the two groups.

**Conclusions:**

Ten genomic loci, containing more than 500 protein coding genes, are identified as more often altered in tumors from early onset versus late onset CRC. Integration of genome and transcriptome data identifies seven novel candidate genes with the potential to identify an increased risk for CRC.

## Background

Less than five percent of all patients diagnosed with colorectal cancers (CRC) carry known genetic germline alterations that predispose to the disease [1]. However, it has been estimated that up to 30% of all CRC patients may carry a genetic risk as suggested by young age at onset, multiple tumors in the same patient, and an excess of individuals with CRC within a family [2,3]. Many studies have tried to identify some of these genetic risk factors, and several recent genome-wide association studies (GWAS) have pinpointed SNP loci on chromosome arms 8q, 10p, 11q, 14q, 15q, 16q, 18q, 19q, and 20p to be associated with CRC [4-10]. Furthermore, a study by Mourra *et al*. [11] showed that microsatellite loci within chromosome arm 14q, known to be deleted in about 30% of all colorectal cancers, were more frequently lost in tumors from early onset patients.

*TP53 *mutations and genomic copy number alterations, as well as other somatic genetic and epigenetic alterations, have been shown to accumulate with the adenoma-carcinoma development in CRC [12-17]. Copy number alterations are typically identified using cytogenetic techniques as G-banding, chromosome-based comparative genomic hybridization (cCGH) and array-CGH (aCGH) [18]. For the most common chromosomal aberrations, which include gains at 7q, 7p, 8q, 11q, 13q, and 20q and losses from 1p, 4p, 4q, 8p, 14q, 15q, 17p, and 18, the time of occurrence in the adenoma-carcinoma sequence has been suggested [16,17,19]. In addition, cCGH has been used to identify DNA sequences that contain predisposing genes, *e.g*. changes in chromosome 19 found in tumors from patients with Peutz-Jeghers syndrome, led to the identification of *STK11 *as the predisposition gene [20]. Other susceptibility loci and genes have been suggested for CRC, typically based on linkage analyses and genome wide SNP analyses [10,21-28].

Array-CGH allows for increased resolution, improves the chromosome dependent method, and thus facilitates detection of small aberrations and fine-tunes the accuracy of breakpoint determination [29]. In order to identify somatic differences and potential susceptibility loci for CRC, we have compared high resolution (385 000 oligo probe array) DNA copy number profile and *TP53 *mutation status in carcinomas from late onset and early onset patients without known hereditary CRC syndromes. These data have further been integrated with corresponding gene expression data for each patient.

## Methods

### Patients and tumor samples

Forty patients diagnosed with CRC, were included in the study. Patient gender and age, and tumor stage and location are shown in Table 1 and Additional file 1. Twenty-three patients with early onset CRC were enrolled from 4 different hospitals in the south-eastern region of Norway. HNPCC, FAP and other known syndromes were excluded after a thorough family and medical history. Seventeen patients with late onset CRC, treated in one of the hospitals, were selected after matching for sex, tumor location and stage.

**Table 1:** Summary of patient data in the early and late onset CRC series.

	Early onset (n = 23)		Late onset (n = 17)		
	n	%	n	%	*p*-value
**Male**	10	43.5	9	52.9	
**Female**	13	56.5	8	47.1	0.75^#^

**Average age**	43.2		78.6		
**Median age**	44.0		79.0		
**min, age**	28		69		
**max, age**	53		87		<0.01^§^

**Dukes' A**	4	17.4	5	29.4	
**Dukes' B**	5	21.7	5	29.4	
**Dukes' C**	10	43.5	5	29.4	
**Dukes' D**	4	17.4	2	11.8 0	0.69^#^

**Proximal colon**	6	26.1	3	17.6	
**Distal colon**	17	73.9	14	82.4	0.71^#^

^# ^Fisher's exact test

^§ ^Student's t-test

Tissue samples were taken and preserved in the operation theatre immediately after resection of the specimen. In the early onset group samples were transferred to tubes with RNA-later RNA Stabilization Reagent (Qiagen, Hilden, Germany), stored at room temperature over night, then transferred to an empty tube and frozen at -80°C for long-term storage. In the late onset group samples were frozen in liquid nitrogen and stored at -80°C until use.

Written informed consent was obtained from all subjects included. The research biobanks are registered according to national legislation and the research project is approved by the Regional Committee for Medical Research Ethics (REK South-East: 1.2005.1629; REK South: 2003, S-02126).

### Nucleic acids isolation

The tumor tissue was manually ground in liquid N_2 _using a mortar and pestle before isolation of DNA. DNA was extracted using a semi-automatic phenol-chloroform extraction method followed by ethanol precipitation in a 340A Nucleic Acid Extractor (Applied Biosystems, Foster City, CA, USA). Ensuring good DNA quality, DNA was measured by using NanoDrop ND-1000 (Thermo Fisher Scientific, Waltham, MA, USA). Samples with OD 260 nm/280 nm >1.7 were included. Thereafter, the DNA was diluted in TE-buffer to a final concentration of 250 ng/μl. The integrity of the DNA was visually inspected on a 1% agarose gel.

RNA was isolated using AllPrep DNA/RNA mini kit (Qiagen). The RNA quality and quantity was measured using the NanoDrop ND-1000 and OD 260 nm/280 nm and OD 260 nm/230 nm was carefully evaluated. Degradation was measured using BioAnalyzer (Agilent Technologies, Santa Clara, CA, USA). All samples included had RNA concentrations above 150 ng/μl, and RNA integrity number (RIN) above 8.0.

### Microsatellite instability analysis

MSI status was determined for all tumors and positive samples were excluded from further analyses, in order to avoid any potential undetected HNPCC patient. Determination of MSI status was performed as described by Wu *et al*.[30], using the Bethesda markers. High degree of microsatellite instability (MSI-H) was defined if two or more markers showed aberrant profile after fragment analysis.

### Array Comparative Genomic Hybridization (aCGH)

From each sample, 1 μg DNA was included in the analysis alongside with gender-matched human reference DNA. Each of the two reference samples, one female and one male reference, consisted of a pool of DNA from normal lymphocytes from four healthy persons. The applied microarray platform, Roche NimbleGen, Human Whole-Genome Array CGH Analysis v1 (Roche Diagnostics, Mannheim, Germany), provides measurements from 385 000 unique genomic loci. The aCGH experimental procedure, as well as raw data pre-processing and normalization, was performed by NimbleGen Systems Inc. at their facility in Iceland [31,32]. For mapping of genomic breakpoints the segMNT v1.1 CGH segmentation analysis algorithm were run in NimbleScan™ software v2.4 (Roche, Basel, Switzerland). A window-size of approximately 60 000 base pairs, calculating the average of 10 probes per window, was further used for analysis.

The female reference pool was hybridized against the male reference pool as a technical control. To determine the threshold for scoring of gain and loss, normalized, log_2_-transformed ratios were used. Based on the variation in autosomal genomic regions, which should not vary between the two reference samples, thresholds for averaged log_2 _ratio data were set to 0.1 and -0.1 for gains and losses, respectively. For the control experiment this implied a false positive rate per genomic loci of .005 and .007 for gains and losses, respectively. As expected when hybridizing female DNA vs. male DNA, gain was seen for the whole of chromosome × whereas loss was seen for the whole of chromosome Y. Furthermore, two samples were run twice on two different arrays. For each doublet of samples the squared correlation coefficient (R^2^) was calculated from all measurements if one or both samples differed from normal copy number. The two samples showed correlation coefficient of 0.84 and 0.75, respectively. For pairs of random samples the correlation coefficient was calculated in the same manner. Median correlation coefficient for ten pairs of random samples was 0.16 (range 0.04 - 0.32).

Statistical differences in frequencies of gains and losses, between the groups of early onset and late onset CRC patients, were calculated using Fisher's exact test. Significance values were for each window/segment calculated based on number of patients with gain or loss compared to no-gain and no-loss, respectively. Affected telomere regions and regions spanning centromeres were not considered as these regions have highly repetitive fragments and may reflect changes that are technical in nature. Also, changes in sex chromosomes were not considered. The number of chromosomal aberrations and the number of breakpoints were calculated as follows: A chromosomal aberration spanning an entire chromosome was recorded as one chromosomal aberration and zero breakpoints; a chromosomal aberration inside a chromosome was recorded as one chromosomal aberration and two breakpoints.

To assign the corresponding chromosome band to specified breakpoints, and identify genes included in given regions we used the UCSC Genome Browser (Mar. 2006 (hg18) assembly, URL: http://www.genome.ucsc.edu) and BioMart genome annotation tool (version 0.7, Ensemble version 53, URL: http://www.biomart.org/), respectively.

### Integration of DNA copy numbers to gene expression data

Genome-wide measurements of mRNA levels were obtained by AB1700 gene expression microarrays (Applied Biosystems, Foster City, CA, USA). Information on sample handling and preprocessing of the raw data will be published elsewhere (Ågesen *et al*., manuscript in preparation). For the integration analysis, we used quantile normalized data with gene-wise centering on the median of the dataset.

**Figure 1:**
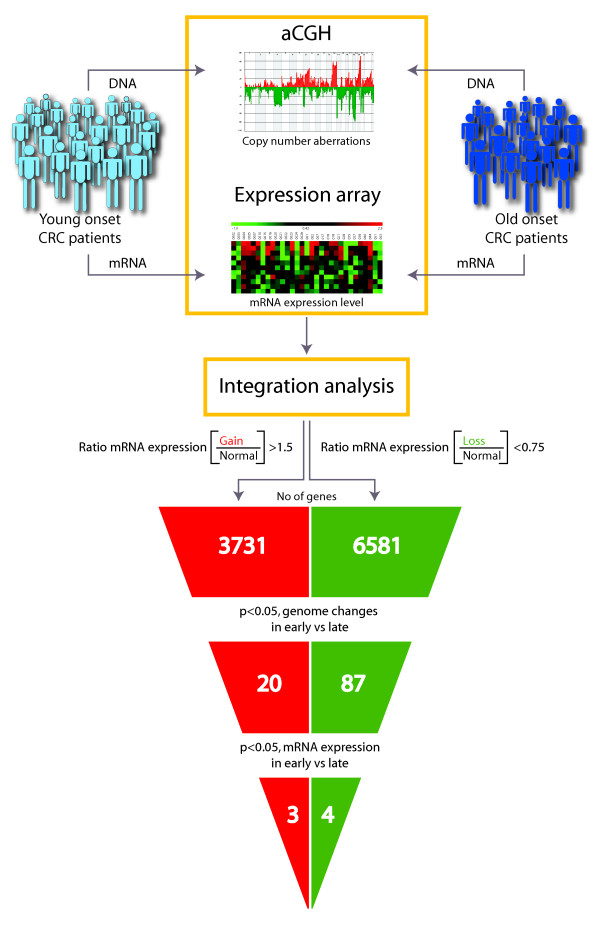
**Study design comparing the tumor genomes of early onset and late onset colorectal cancer patients**. Tumor samples from early onset and late onset CRC patients were analyzed with aCGH (DNA/genome level) and the data were integrated with the mRNA expression (RNA/transcriptome level) of the same samples. About 10 000 genes show corresponding DNA and RNA level. Exclusion of genes located outside chromosomal regions with statistically significant difference between the two patient groups resulted in 107 genes, summarized in Additional file 6. Further, exclusion of genes whose mRNA expression levels were not statistically significant between patients groups resulted in a short list of seven potential predisposing genes, as summarized in Table 3.

Each measurement on the mRNA expression array was related to the DNA copy number status of the corresponding genomic loci. A visual basic (VBA) script was written to automate the analyses and to perform integrated statistics genome-wide on a gene-by-gene and locus-by-locus manner. For each gene, and for gain, loss and normal copy number independently, the median of the expression levels for the samples with the DNA copy number changes was divided by the median of the expression levels for the samples with normal copy number. To indicate genes with concomitant gain and over-expression, we set a threshold at 1.5, and genes with concomitant loss and under-expression were indicated when the value was below 0.75. Furthermore, the expression levels for genes located in regions with statistically significant difference in gain or loss in the early onset compared to the late onset CRC samples, were evaluated using Independent samples T-test in SPSS. Annotations of genes are used according to AB1700 annotation file (version; 20060930_ab1700_human, Applied Biosystems). Overview of the study is given in Figure 1.

### *TP53 *mutation analysis

The total protein coding region of *TP53 *was amplified in a multiplex PCR reaction containing five distinct PCR fragments by using flanking intronic primers with M13 tails. Multiplex PCR kit was used as recommended by the vendor (Qiagen, Hilden, Germany). If initial multiplex PCR reaction did not succeed, the same fragments were amplified separately using each of the primer pairs. Primer and fragment details are described in Additional file 2. After visual inspection of quality and quantity on a polyacrylamide gel, the product was purified using Exo-SAP-IT (USB Corporation, Cleveland, OH, USA). Sequencing of the purified products was performed in both 5'- and 3' directions using BigDye Terminator v3.1 kit (Applied BioSystems, Foster City, CA, USA). Primers for the sequencing reaction were identical with those used in the initial PCR for fragments amplified in multiplex reactions. M13-primers were used in sequencing reactions of singleplex PCR products. The resulting sequence product was further purified using multiscreen plates, (MilliPore, Billerica, MA, USA) with Sephadex™ G-50 Superfine (GE Healthcare, Chalfont St. Giles, UK), and subjected to sequencing at a 3730 DNA Analyzer (Applied Biosystems, Foster City, CA, USA). If a mutation was detected, a new independent PCR product was subjected to sequencing to confirm our finding.

## Results

### Genomic profile of colorectal carcinomas

**Figure 2:**
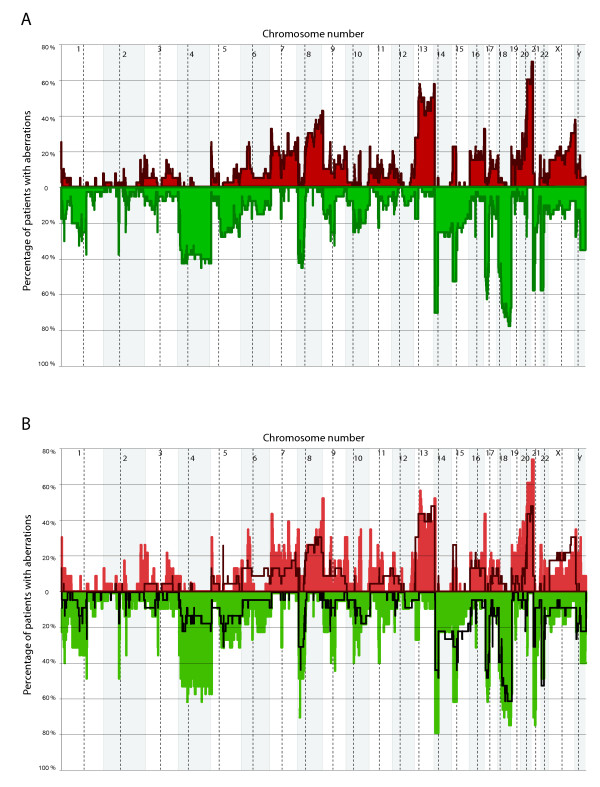
**Genomic profile across all chromosomes from 40 tumor samples**. **A) **Copy number profile across all 40 colorectal carcinomas, including early onset (n = 23) and late onset (n = 17) tumors. Percentage of samples with gains is shown in red and losses in green. Gains and losses in the short arm of acrocentric chromosomes are not considered and further discussed. The centromere positions are indicated by dashed lines, and changes recorded at the p-arms and q-arms are shown to the left and right of the dashed lines, respectively. **B) **Copy number profiles of early onset (solid) as compared to late onset (line) colorectal carcinomas.

The profile of DNA copy number gains and losses across all samples is shown in Figure 2A. Overall, across the 40 MSS colorectal samples the median number of chromosomal aberrations was 31.5 (range 11-93), and the median percentage of probes showing normal copy number was 78% (range 48%-99%).

Gains at 8q, 13q and 20q and losses from 4p, 4q, 8p, 17p, 18p and 18q were most frequent (range 43%-78%). Aberrations in the p-arm of acrocentric chromosomes were not considered as these most probably reflect technical challenges. The most frequent loss, 18q22.1-22.3 affecting ~10 Mbps, was found in 78% of all samples. This region comprises 34 protein coding genes. The region most often gained, found in 70% of the tumors, were a ~7.5 Mbps region at 20q13.31-13.33. This region contains 10 miRNAs and 100 genes. One of the most commonly affected chromosomes, chromosome 8, showed losses from the p-arm in 65% of all tumors and gains at the q-arm in 43%. For the majority of the samples, the breakpoint between gain and loss at chromosome 8 was located in the p-arm, about 5 Mbps away from the centromere, as shown in Additional file 3.

### Genomic profiles of early onset versus late onset colorectal carcinomas

The average fraction of the genome with aberrant copy number was similar in the early onset and late onset CRC series, 26% versus 22%, respectively (*P *= 0.53). However, both the number of chromosomal aberrations and thus the number of breakpoints differed significantly between the groups (*P *= 0.02 and *P *= 0.01, respectively). Chromosome regions with aberrations are listed in Table 2. Overview of aberrations from each patient sample is shown in Additional file 4.

**Table 2:** Genomic loci with statistically significant copy number variation between early and late onset colorectal cancers.

Chromosome	Band	Mbps from pter	Size (Mbps)	p-value (average)
**Gain**				
2	q35	217.2	3.2	0.03
5*	q13.2	69.5	0.3	0.03
10	q21.3-22.1	70.9	2.4	0.02
10	q22.3	79.5	1.5	0.01
19	q13.2-13.31	44.6	3.9	0.02

**Loss**				
1	p31.3	61.5	0.3	0.02
1	q21.1	145.02	0.6	0.02
2	q21.2	132.78	0.1	0.02
4	p16.1	8.88	0.7	0.02
4	p14	39.6	1.0	0.05
4	q21.3-22.1	88.02	0.4	0.02
4	q22.1-28.2	88.44	41.1	0.04
4	q28.2-28.3	129.96	2.0	0.05
4	q28.3	132.96	0.3	0.03
10	p11.1	38.94	0.1	0.03
19	p12	20.52	0.3	0.03

*Aberration is significantly more often present in the late onset than the early onset patients.

**Figure 3:**
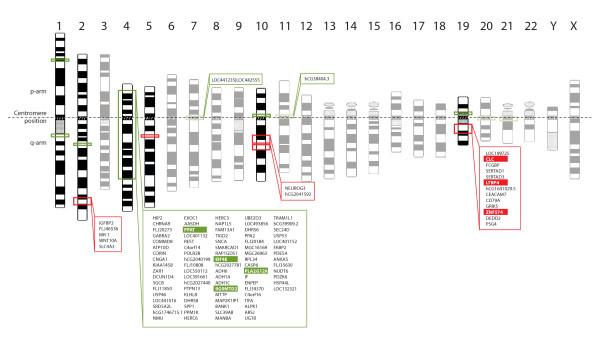
**Genes mapping to the chromosome bands with copy number aberrations associated with early onset CRC**. Chromosomal sites gained or lost with statistical significance in the early compared to the late onset tumor group are shown in red or green boxes, respectively. Chromosomes with no aberrations or aberrations in centromere regions only, are shaded. Genes which have expression levels corresponding to chromosomal aberration, and concurrently located within the statistically significant regions are indicated within the boxes. Genes which also have significantly different expression level between early onset and late onset groups are further marked with green or red background.

A plot of gains and losses along a genome axis for each of the two patient groups shows distinct differences (Figure 2B). Statistical calculations suggested that 3.2% of the genome was significantly different between the two groups. This includes 16 different regions at 6 unique chromosomes, spans a total of 58.3 Mbps, and affects 107 genes, (Figure 3). Four regions with statistical significant difference were spanning centromere regions and were excluded from further analysis. Only 0.3 Mbps of these changes (one region) were more often observed in late onset cancers than in the early onset group (Table 2). The parts of the genome that exhibited significant difference between the two tumor groups contains, among others, 574 protein coding genes, 44 miRNA, and 47 pseudogenes, as listed in Additional file 5.

Overall, the smallest region of overlap (SRO) of losses with the most statistically significant difference between early onset and late onset patients (*P *= 0.003) was located 0.24 Mbps adjacent to the centromere in chromosome 4. Only one miRNA maps to this position. Fourteen of the 23 (61%) early onset CRC patients, as compared to two of 17 (12%) late onset patients, displayed loss in this region. The most significant SRO of losses containing protein coding genes was located at 1q21.1 (*P *= 0.01). This position contains one protein coding gene, namely *PRKAB2*, encoding a regulatory subunit of the AMP-activated protein kinase (AMPK). Ten of 23 (43%) early onset CRC patients, and only one of 17 (6%) late onset patients, displayed loss of this locus.

Copy number differences between the patient groups were found for large parts of chromosome 4 (4p16.1-q28.3) (Figure 2B). In total, the regions on chromosome 4 with significant differences between the groups contained 258 protein coding genes, 36 miRNAs and 27 pseudogenes.

Two sequence stretches within 10q22.3 and 19q13.31 were gained in 8 of 23 patients in the early onset group versus none among the 17 late onset patients (*P *= 0.01). The 10q region contains four protein coding genes, *SFTPA2, ZCCHC24, PPIF *and *ZMIZ1*, whereas the 19q region includes only *PSG3 *(pregnancy-specific beta-1-glycoprotein 3). At chromosome 19 a 4 Mbps gained region (19q13.2-q13.31) was significantly more frequent in the early onset group. This comprises 103 protein coding genes and three miRNAs. The protein coding genes include, among others, a group of genes in the carcinoembryonic antigen (CEA) family.

Only one region, spanning 0.3 Mbps at 5q13.2, was gained more often in late onset patients (5 of 17) compared to early onset patients (none of 23). The region contains 4 putative protein coding genes, β-clucoronidase-like protein SMA5, β-clucoronidase-like protein SMA4, ENSG00000205565, and ENSG00000197370.

### Integrated genome and transcriptome analysis

**Figure 4:**
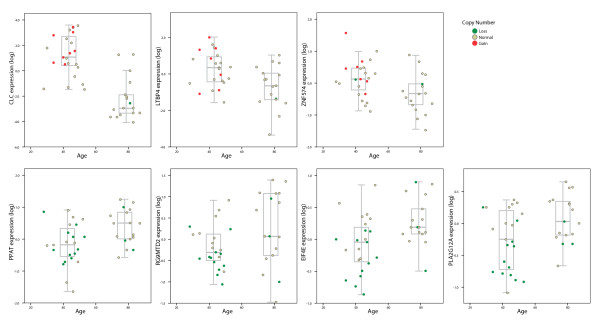
**mRNA expression levels of a short list of seven candidate genes for early onset CRC**. Log transformed mRNA expression values plotted against age. Red bullets indicate copy number gain, green bullets indicate loss and grey bullets indicate normal copy number. Box plots for each of the patient groups are indicated for each gene.

An integration analysis of the DNA copy number data and mRNA expression data revealed that 37% (~10 900) of the genes had corresponding DNA copy number and RNA expression levels. Among these, 107 genes were located in genomic regions with significantly different frequency of copy number changes between the two tumor groups (Additional file 6). When sorting these 107 genes based on fold-change of expression levels between the groups, *CLC*, *CEACAM7*, *FCGBP*, hGC1641029.5 (Probe ID:218092) and *CD79A *were top five for gains, and the top five list for losses included LOC391661(Probe ID:152373), *ARSJ*, *ADH6*, *SPP1 *and *BANK1*. From the 107 genes, those with significantly different mRNA expression levels between the early onset and late onset groups were identified. The resulting strict gene list included seven genes (Table 3 and Figure 3). Of these, three genes (*CLC*, *LTBP4 *and *ZNF574*), all within chromosome band 19q13.2, were concomitantly gained and up-regulated, and four genes (*PPAT*, *EIF4E*, *RG9MTD2 *and *PLA2G12A*) were lost and down-regulated. The *PPAT *gene is located in 4q21, *EIF4E *and *RG9MTD2 *in 4q23 and *PLA2G12A *in 4q25. The expression levels for each gene are shown in Figure 4.

**Table 3:** Genes/Probe IDs showing statistically significant different DNA copy number and mRNA expression in early versus late onset colorectal cancers.

					Patients with chromosomal aberrations	
Copy number aberration	Gene/Probe ID	Chromosome band	Mbps§ from pter	Size# (Kbps)	Early onset (n = 23)	Late onset (n = 17)
**Gain**	CLC/118354	19q13.2	44.9	6.8	7	0
	LTBP4/162965	19q13.2	45.8	30.6	6	0
	ZNF574/130858	19q13.2	47.3	11.2	6	0

**Loss**	PPAT/156570	4q12	57.1	42.3	12	3
	EIF4E/195966	4q23	100.2	22.1	12	3
	RG9MTD2/125412	4q23	100.8	16.9	12	3
	PLA2G12A/106464	4q25	111.0	16.5	12	3

^§^Gene start position, indicated as Mbps from pter

^#^Gene size, indicated in Kbps

### *TP53 *mutations and genetic complexity

Mutation status of *TP53 *was evaluated for all protein coding exons. Sixty-five percent of the samples were mutated, with no significant difference being observed in the early onset group compared to the late onset group (Additional file 1). Five percent of the mutations were located outside the DNA binding domain of *TP53*. A region frequently lost at 17p comprises the p13.1 band where the *TP53 *gene is encoded. Loss in this region was observed in 55% of the tumor samples, also with no frequency difference between the early onset and the late onset group. Overall, 80% of the samples displayed mutation in *TP53 *and/or DNA copy number aberration of 17p13.1. Both loss of 17p13.1 and mutation in *TP53 *were independently associated with an increased number of chromosomal aberrations (*P *< 0.01 and *P *< 0.01, respectively, and *P *= 0.03 for loss and mutation combined). This difference was still significant when comparing mutated (n = 9) and wild type (n = 8) samples with normal copy number in 17p13.1 only (*P *= 0.01).

The concurrent loss and reduced expression of *PPAT*, *EIF4E*, *RG9MTD2*, and *PLA2G12A*, were significantly correlated to *TP53 *mutations (*P *= 0.006) in the tumors from the early onset patients. The same trend was seen for tumors with concurrent gain and elevated expression of *CLC*, *LTBP4 *and *ZNF574*, though not statistically significant, (*P *= 0.17).

## Discussion

In Norway, around 5% of all patients with CRC are diagnosed before the age of 55. However, the incidence rate increases with increasing age, and the median age at diagnosis is 70 years. Individuals with early onset of CRC may be carriers of gene variants causing an increased risk for disease, but most of these predisposition genes remain to be identified. The present study demonstrates genetic aberrations that are associated with early onset CRC. Compared to the current literature, the number of patients (n = 40) is fairly high, and the number of measurements provided is very high.  To our knowledge no previous study has at this resolution level compared the genomic changes between tumors from patients with ~20 year difference in onset.  Methods for genome-wide assessment of DNA copy number alterations in most studies published to date are generally of low to medium resolution. The introduction of aCGH has increased the resolution hundreds of times, and with the microarray platform used in this study the total number of probes is 385 000, giving an average probe interval of 6000 bases.  In addition, measurements of mRNA expression levels from the same samples have allowed for a short list of candidate genes.

### Genomic alterations in colorectal cancers

The overall DNA copy number profile for CRC found in the present study is in line with previous reports [19], suggesting that the series used are representative for microsatellite stable CRCs. The profile includes frequent gains at 8q, 13q, and 20q and losses from 4p, 4q, 8p, 17p, 18p and 18q.

As expected the most common loss and gain were found at 18q and 20q, respectively. The SROs in these chromosome arms contains a number of protein coding genes, including well known critical cancer genes as *BCL2 *(18q22), *DCC *(18q21) and *AURKA *(20q13). The latter is known to be important for normal chromosome segregation, observed as over-expressed in cancers with chromosomal instability (CIN) [33]. Also present in 20q13 are several members of the SERPIN gene family, including *SERPINB5*, positively regulated by TP53 [34]. Furthermore, one of the 10 miRNAs in this region is the MIR646 (hsa-miR-646), reported to be related to colorectal cancer (The miR-ontology database, http://ferrolab.dmi.unict.it/miro) [35].

In CRC, aberrations at chromosome 8 are commonly reported as loss in 8p and gain in 8q [36]. Our data shows that the breakpoint is located on the p-arm, approximately five Mbps from the centromere (Additional file 3). This is in accordance with findings from others [37,38]. This clearly shows that the resolution of aCGH is crucial for a more exact evaluation of breakpoint regions. Loss of gross parts of both arms of chromosome 4 is frequently reported in several cancers [39-44], and the location of tumor suppressor genes important for tumorigenesis in this region is plausible [45]. In this study we observed on average 40% of losses in chromosome 4.

### Differences in genome profile between early onset and late onset CRC

In the early onset sample series, the number of chromosomal aberrations and the number of breakpoints were significantly higher than in the late onset sample series. However, the percentage of measurements showing aberrations does not differ significantly. This indicates that about the same amount of DNA is affected by copy number changes in the two series, but the number of events is larger in the early onset group compared to the late onset samples, which again suggests increased genome instability in the early onset tumors.

Fifteen loci at five unique chromosomes were statistically more often altered in the tumors of the early onset group compared to the late onset group (Table 2). The gained region at chromosome 19 with statistically significant difference between the two patient groups spanned 4 Mbps. This region comprises more than one hundred protein coding genes, among them a group of genes in the carcinoembryonic antigen (CEA) family. Genes in this family encode pregnancy-specific beta-I-glycoproteins and carcinoembryonic antigen-related cell adhesion molecules. Also, 19q13.2-q13.31 contains three microRNAs, of which one, the MIR641 (hsa-miR-641), is known to be associated with colorectal cancer [35]. Chromosome 19 is notable for the highest gene density of all human chromosomes, large clustered gene families and high GC content, indicating biological and evolutionary significance [46]. In a recent GWAS study, a region on 19q13.1 was identified as a putative susceptibility locus for CRC [10]. This region is 6.4 Mbps proximal to the region we have identified at 19q13.2-q13.31.

In the present study six smaller regions at chromosome 4, comprising ~70 Mbps in total, were identified with statistical significant losses in the early onset group compared to the late onset patients (Table 2). In total ~250 genes and 30 miRNAs are located in the affected regions. Even though deletions in chromosome 4 is a common feature in CRC, and known cancer genes such as *KIT*, *EGF *and *FGF2 *are situated here, none has yet been verified as predisposing for early onset or hereditary CRC.

Chromosomal loci previously suggested to contain genes that may predispose to CRC [4-10] are not the same as the ones found in the present study. This may best be explained by the difference in inclusion criteria; whereas the GWAS-studies have mainly been performed in samples from individuals from high-risk families, the present study selected patients with young age at diagnosis of disease. Mourra *et al*. reported a statistically higher frequency of deleted 14q loci in patients <50 years at primary diagnosis versus patients >50 years at diagnosis [11], but this could not be confirmed in the present study.

### Integration of DNA copy number and mRNA expression data

The integration analysis of genome copy number data with mRNA expression data identified a short list of 7 target genes differing between the early- and late onset groups. The three gained and up-regulated genes, *CLC, LTBP4*, and *ZNF574*, were altered in the same tumors. The *CLC *gene differentiates the tumor groups the most with regard to mRNA expression levels (*P *= 0.001). The CLC protein (Charcot-Leyden crystal protein/Galectin-10) is a lysophospholipase in the galectin super family of proteins, normally expressed in eosinophils and basophils, associated with inflammation and some myeloid leukemia [47]. However, data on CLC in cancer is sparse.

The LTBP4 protein (latent TGF-β binding protein 4) has structural roles in the extracellular matrix as well as its participation in the TGFβ-pathway. TGFβ-pathway components are commonly altered in cancer in general, and in colorectal cancer in particular. On of them, *SMAD4*, cause juvenile polyposis when mutated in germline cells. It is localized at chromosome 18q21 together with *SMAD2*, in a region frequently deleted in colorectal cancers. *TGFβRII *mutations are found in ~30% of colorectal cancers and are considered the main mechanism of inhibition of the TGFβ-pathway. Although mutations in *TGFβRI *are rare, an association between the TGFBR1*6A-polymorphism and cancer has been reported [48]. Together with LAPs (latency-associated proteins), LTBP4 it is involved in the assembly, secretion and targeting of TGFβs [49]. TGF-β has an important, complex and somewhat dual role in the normal cell. Downstream effects are as diverse as promotion of migration, adhesion and differentiation on one hand, to inhibition of growth, cell-cycle control and apoptosis on the other [50]. As opposed to the present results, others have reported a suppression of LTBP4 to cause cancer [51,52]. An increase in LTBP4 expression might potentially cause colorectal cancer development. Elevated levels might increase deposition of TGFβ in extracellular matrix, thus hindering TGFβ to bind to the receptor, and thereby prevent tumor suppressive downstream reactions such as apoptosis, and growth arrest.

The concomitantly lost and down-regulated genes *PPAT*, *EIF4E*, *RG9MTD2*, and *PLA2G12A *are located on different bands of chromosome 4. All these four candidate genes and potential tumor suppressor genes had a combined genomic loss and mRNA under-expression in the same patients, presumably associated with monosomy. *PLA2G12A *is a member of the secreted PLA2s (sPLA2) in the phospholipase A2 family of proteins, found to be involved in tumorigenesis [53-55]. The enzymatic activity of PLA2s is to hydrolyze the fatty acid from membrane phospholipids, which are further metabolized and forms eicosanoids and bioactive lipid mediators [56]. *PLA2G12A *has been reported to be highly expressed in normal and tumor tissues from the colon [53], which suggests that reduced expression, as reported in this study, might contribute to an oncogenic transformation in a subset of early onset CRCs. The protein EIF4E is a translation initiation factor, and mRNA expression is reported to be elevated in many human cancers [57]. mRNA levels are reported to increase during tumor formation and progression of colorectal cancers [58]. In our study the expression of *EIF4E *was found to be reduced in the early onset group compared to the late onset group, which may indicate that early onset cancers develop in an EIF4E independent manner.

### *TP53 *mutations and genomic complexity

*TP53 *mutations are associated with CIN tumors. The *TP53 *gene was sequenced to confirm an equal representation of mutations in the two age groups, to ensure that the series were unbiased. The seemingly high frequency of 65% mutations reported here is most likely due to the fact that all protein coding exons in *TP53 *were analyzed, and that only microsatellite stable tumors are included in the study. The samples with loss in 17p13.1 had significantly more aberrations throughout the whole genome, compared to the samples with normal copy number or gain. This is as expected, partly because loss of 17p is associated with CIN phenotype, and partly because one expects any sample with aberration in a given position to have more aberrations globally. Furthermore, a statistically significant difference in percentage of aberrations was seen when comparing mutated (n = 27) to wild type (n = 13) samples, irrespective of DNA copy number in the region where *TP53 *is located. Samples with no aberrations in *TP53 *showed a lower degree of genetic complexity measured as the copy number level throughout the genome. This clearly indicates that non-functional *TP53*, regardless of how it is inactivated, plays an important role in tumorigenesis associated with CIN.

Interestingly, loss of 1q21 containing the AMP kinase subunit β2 (*PRKAB2*), which was recently shown by the Arnold Levine laboratory to be regulated by TP53 [59], are more often lost in tumors with wild type *TP53 *than in those with mutated *TP53 *(*P *= 0.08).

### Cancer susceptibility and genetic pathways

Our finding that carcinomas from early onset patients present a high number of chromosomal aberrations and breakpoints is comparable with the concept that they have inherited a germline mutation in a gene relevant for chromosome stability, with loss of the second allele occurring somatically. Relevant examples for such a relationship are the complex pattern of copy number changes observed in breast carcinomas associated with germline *BRCA1 *mutations (when compared with sporadic breast carcinomas), and the microsatellite instability observed in colorectal carcinomas from Lynch syndrome patients [60-62]. The data we present here highlight possible candidate susceptibility loci that complement other studies using different strategies [5,6,10,21-25,63-66]. Alternatively, the changes we have found more frequent in tumors from early onset patients, may reflect changes as a result of somatic mutations related to a yet uncovered germline mutation.

## Conclusions

This is the first study that has identified distinct tumor genome profiles in early onset and late onset CRC patients at 6 Kbps resolution level, combined with the corresponding mRNA expression profile. Sixteen genomic loci containing more than 500 coding genes were identified as preferentially altered in cancers from early onset patients when compared to late onset patients. Finally, by integration analysis with gene expression data from the same samples, we identified a short list of seven candidate genes as potentially predisposing to early onset CRC. Further studies are warranted to find out if early onset CRC is caused by inherited low penetrance alleles or germline mutations in these or other genes.

## Competing interests

The authors declare that they have no competing interests.

## Authors' contributions

MB and THÅ have performed the experimental analyses. MB has drafted the manuscript, made all figures and performed statistical- and computational analysis. ETE, MAM, MHV, AN and the INFAC-study group have provided the tumor series and the patient information. MRT participated in the evaluation of results and in the manuscript preparation. ETE, AN, and RIS participated in the study design. RIS supervised the bioinformatic analyses, participated in evaluation of results and in the manuscript preparation. RAL conceived the study, was main responsible for the study design and study coordination, participated in evaluation of results and in manuscript preparation. All authors have contributed to the writing and have read and approved the final version of the manuscript.

## Supplementary Material

Additional file 1 Summary of clinical data for all patients included in the study. Click here for file

Additional file 2 **Primer details for *TP53 *mutation analysis**. Details of the fragments amplified for *TP53 *analysis. Both 5'- and 3' primers have M13 tails, indicated in capital letters. Click here for file

Additional file 3 **Detailed profile of gains and losses in chromosome 8**. Percentage of gains and losses along chromosome 8 for all colorectal carcinomas in the presented study. Centromere position is indicated by solid line, breakpoint region by dashed line. Click here for file

Additional file 4 **Overview of aberrant genomic regions in each carcinoma**. Detailed listing of regions displaying gain or loss for each patient sample. Click here for file

Additional file 5 **Loci within the chromosome bands that show statistically different copy number changes in colorectal carcinomas between patient groups**. Protein coding genes, miRNAs and pseudogenes in all regions with statistically significant difference in copy number between patient groups are extracted by using BioMart with Ensemble version 53. Click here for file

Additional file 6 **Genes that show corresponding mRNA expression and DNA copy number changes in early onset CRCs**. Genes (with respective probe IDs) with mRNA expression levels corresponding to DNA copy number, also situated in regions with statistically significant difference in early onset compared to late onset patients are listed. The expression ratio is calculated as median expression in samples with gain or loss divided by median expression in samples with normal copy number. The number of tumors with copy number aberration in the early onset and late onset groups are indicated, as well as the *P*-value. The resulting genes are primarily sorted by increasing *P*-value, based on aCGH-analysis alone, followed by a sort based on the expression-ratio. Click here for file
