# Supplementary figures and images for: Distinct high resolution genome profiles of early onset and late onset colorectal cancer integrated with gene expression data identify candidate susceptibility loci
Source: Mol Cancer. 2010 May 6;9:100. doi: 10.1186/1476-4598-9-100 (PMC2885343; doi:10.1186/1476-4598-9-100)

Profile of chromosome 8 for all samples in the present study

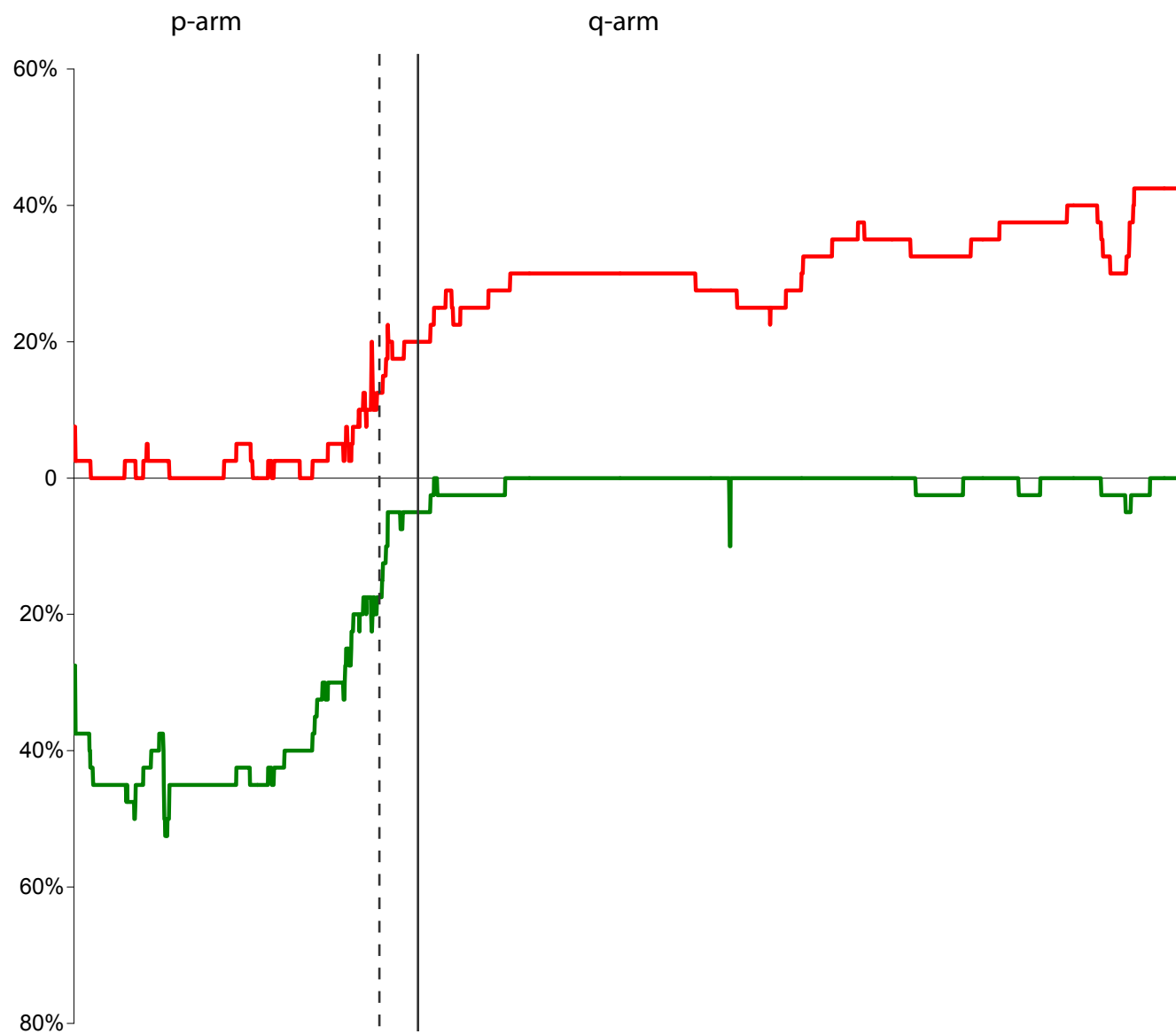

Supplement: Additional file 3 — Detailed profile of gains and losses in chromosome 8. Percentage of gains and losses along chromosome 8 for all colorectal carcinomas in the presented study. Centromere position is indicated by solid line, breakpoint region by dashed line. [file 1476-4598-9-100-S3.PDF]
